# Complete Genome Sequence of *Aureimonas* sp. Strain OT7, Isolated from Human Skin

**DOI:** 10.1128/MRA.00024-21

**Published:** 2021-03-04

**Authors:** Munkhtsatsral Ganzorig, Kyoung Lee

**Affiliations:** a Department of Bio Health Science, Changwon National University, Changwon, Republic of Korea; Loyola University Chicago

## Abstract

*Aureimonas* sp. strain OT7 was isolated from human skin. This strain can grow on Triton X-100. Here, we present the complete whole-genome sequence of this species, which has one chromosome of 4,181,223 bp (G+C content, 65.05%). Analysis of the *Aureimonas* sp. strain OT7 genome sequence indicated potential for autotrophic growth.

## ANNOUNCEMENT

Strains of Gram-negative Aureimonas altamirensis have been isolated from diverse environments and human skin (1, 2) and have been also implicated in human infections (3, 4). In most cases, identification of the species of the clinical isolates has been based on the 16S rRNA gene sequence identities. Two genome assemblies of *A. altamirensis* from strains DSM 21988^T^ and ON-56566 are publicly available in the NCBI database. These strains were isolated from a subterranean environment and the blood culture of a patient with cellulitis, respectively (1, 4). In this study, we isolated *Aureimonas* sp. strain OT7 from human skin for its ability to grow on Triton X-100, a nonionic detergent.

A swab sample from the skin of the back of a male college student’s ear was directly streaked onto minimal salts basal medium (MSB) agar (5) containing 0.5% Triton X-100 as a source of carbon and energy. The agar medium was aerobically incubated for 1 week at 28°C for growth. One strain, named OT7, was further purified by streaking on nutrient broth agar (Difco Co.) with 0.1% sodium pyruvate and then incubated at 28°C for 2 days. This strain was deposited in the Korean Collection for Type Cultures as KCTC 82307. Ethical approval for subject sampling was granted by the institutional review board of Changwon National University. The 16S rRNA gene of strain OT7 was amplified from lysed colony material by PCR with the universal primers 27F and 1492R (6) using *Taq* DNA polymerase (Bioneer, Republic of Korea). Following Sanger sequencing, a similarity search with the sequences of type strains using BLASTn v.2.11.0 with default settings in NCBI (rRNA/ITS databases) (7) revealed the highest sequence identity to *A. altamirensis* DSM 21988^T^ (99.47%). Strain OT7 has been further characterized by genomic sequencing.

For DNA extraction, cells were cultured in a flask on nutrient broth with 0.1% sodium pyruvate for 48 h at 28°C with shaking at 140 rpm. Total genomic DNA was purified using the phenol extraction method (8). The DNA was quantified with NanoDrop UV-visible spectroscopy (S-3100; Sinco, Republic of Korea) and spectrofluorimetry (Qubit 4; Thermo Fisher Scientific, USA). For genomic DNA sequencing, 5 μg genomic DNA was sheared into 20-kb fragments using a g-TUBE device (Covaris, USA) and purified using the AMPure XP bead purification system (Beckman Coulter, USA) according to the manufacturers' protocols. A DNA library with an insert size of 20 kbp was prepared using the SMRTbell template prep kit 1.0 (PN 100-259-100) and the PacBio DNA/polymerase binding kit P6 according to the manufacturers’ protocols. The Pacific Biosciences RS II platform with SMRT Analysis 2.3.0 p4 software with default settings was utilized for library sequencing (9), read filtering, and genome assembly at DNA Link Co. (Seoul, Republic of Korea). With a minimum polymerase read quality of 0.8, the OT7 genome yielded 105,216 reads encompassing 1,136,303,269 bp. The postfilter with a length cutoff of 1,000 bp yielded 39,455 sequencing reads with 1,119,510,078 bp and an *N*_50_ value of 15,790 bp. Subsequently, the clean reads were *de novo* assembled using the Hierarchical Genome Assembly Process (RS_HGAP3_Assembly) workflow, which included consensus polishing using Quiver. Gene predictions and annotations were provided by NCBI using the NCBI Prokaryotic Genome Annotation Pipeline 4.13 (10).

The final genome assembly of *Aureimonas* sp. strain OT7 consists of one circular chromosome of 4,181,223 bp (G+C content, 65.05%), with 267.7× contig sequencing coverage. The chromosome contains 3,875 protein-coding sequences, three copies of rRNA genes (5S, 16S, and 23S), 51 tRNA coding regions, and 4 noncoding RNA (ncRNA) genes. The genome sequence of strain OT7 shared BLAST average nucleotide identity (ANIb) values, as determined by the JSpeciesWS server (11), of 91.20% and 97.46% with *A. altamirensis* DSM 21988^T^ (GenBank accession number GCA_001463885.1) and *A. altamirensis* ON-56566 (GCA_000800175.1), respectively. Based on the above 95% ANIb species-level thresholds (11), strains OT7 and ON-56566 belong to the same species clade, which is distantly related to the species *A. altamirensis*. This result deserves further taxonomic studies on these strains and clinical isolates. The *Aureimonas* sp. strain OT7 genome sequence contained genes coding for the glyoxylate cycle, required for Triton X-100 degradation by Pseudomonas nitroreducens TX1 (12), genes related to the Calvin-Benson cycle, and RuBisCO genes for autotrophic growth, like other skin-derived bacterial isolates (13, 14).

### Data availability.

The complete genome sequence of *Aureimonas* sp. strain OT7 was deposited in GenBank under the accession number CP062167.1. The BioProject, BioSample, and SRA accession numbers are PRJNA665798, SAMN16268744, and SRX9198886, respectively.

## References

[1] Jurado V, Gonzalez JM, Laiz L, Saiz-Jimenez C. 2006. *Aurantimonas altamirensis* sp. nov., a member of the order *Rhizobiales* isolated from Altamira Cave. Int J Syst Evol Microbiol 56:2583–2585. doi:10.1099/ijs.0.64397-0. 17082395

[2] Cosseau C, Romano-Bertrand S, Duplan H, Lucas O, Ingrassia I, Pigasse C, Roques C, Jumas-Bilak E. 2016. Proteobacteria from the human skin microbiota: species-level diversity and hypotheses. One Health 2:33–41. doi:10.1016/j.onehlt.2016.02.002. 28616476 PMC5441325

[3] Luong M-L, Békal S, Vinh DC, Lauzon D, Leung V, Al-Rawahi GN, Ng B, Burdz T, Bernard K. 2008. First report of isolation and characterization of *Aurantimonas altamirensis* from clinical samples. J Clin Microbiol 46:2435–2437. doi:10.1128/JCM.00337-08. 18495852 PMC2446920

[4] Eshaghi A, Shahinas D, Patel SN, Kus JV. 2015. First draft genome sequence of *Aureimonas altamirensis*, isolated from patient blood culture. FEMS Microbiol Lett 362:fnv016. doi:10.1093/femsle/fnv016. 25714548

[5] Stanier RY, Palleroni NJ, Doudoroff M. 1966. The aerobic pseudomonads: a taxomonic study. J Gen Microbiol 43:159–271. doi:10.1099/00221287-43-2-159. 5963505

[6] Frank JA, Reich CI, Sharma S, Weisbaum JS, Wilson BA, Olsen GJ. 2008. Critical evaluation of two primers commonly used for amplification of bacterial 16S rRNA genes. Appl Environ Microbiol 74:2461–2470. doi:10.1128/AEM.02272-07. 18296538 PMC2293150

[7] Altschul SF, Gish W, Miller W, Myers EW, Lipman DJ. 1990. Basic local alignment search tool. J Mol Biol 215:403–410. doi:10.1016/S0022-2836(05)80360-2. 2231712

[8] Ausubel FM, Brent R, Kingston RE, Moore DD, Seidman JG, Smith JA, Struhl K (ed). 1990. Current protocols in molecular biology. John Wiley & Sons, New York, NY.

[9] Lee K, Ganzorig M, Jung JY, Badaya SK, Lim JY. 2019. Complete genome sequence of *Kocuria indica* CE7, isolated from human skin. Microbiol Resour Announc 8:e00607-19. doi:10.1128/MRA.00607-19. 31296687 PMC6624770

[10] Tatusova T, DiCuccio M, Badretdin A, Chetvernin V, Nawrocki EP, Zaslavsky L, Lomsadze A, Pruitt KD, Borodovsky M, Ostell J. 2016. NCBI Prokaryotic Genome Annotation Pipeline. Nucleic Acids Res 44:6614–6624. doi:10.1093/nar/gkw569. 27342282 PMC5001611

[11] Richter M, Rossello-Mora R, Oliver Glockner F, Peplies J. 2016. JSpeciesWS: a Web server for prokaryotic species circumscription based on pairwise genome comparison. Bioinformatics 32:929–931. doi:10.1093/bioinformatics/btv681. 26576653 PMC5939971

[12] Nguyen TN, Yeh C-W, Tsai P-C, Lee K, Huang S-L. 2016. Transposon mutagenesis identifies genes critical for growth of *Pseudomonas nitroreducens* TX1 on octylphenol polyethoxylates. Appl Environ Microbiol 82:6584–6592. doi:10.1128/AEM.01907-16. 27590807 PMC5086552

[13] Lim JY, Ganzorig M, Huang S-L, Lee K. 2019. First complete genome sequence of *Haematobacter massiliensis* OT1 (chromosome and multiple plasmids), isolated from human skin. Microbiol Resour Announc 8:e00292-19. doi:10.1128/MRA.00292-19. 31048379 PMC6498234

[14] Lim JY, Hwang I, Ganzorig M, Pokhriyal S, Singh R, Lee K. 2018. Complete genome sequence of *Paracoccus yeei* TT13, isolated from human skin. Genome Announc 6:e01514-17. doi:10.1128/genomeA.01514-17. 29348361 PMC5773746

